# Should the Doctor Tell?

**Published:** 1917-12-22

**Authors:** 


### The Hospital

The Workers' Newspaper of Administrative Medicine and Institutional Life, Administration, National Insurance and Health.

No. 1646, Vol. LXIII. SATURDAY, DECEMBER 22, 1917. PRICE ONE PENNY

### SHOULD THE DOCTOR TELL?

In our issue of December 8 (p. 207) a correspondent, Dr. G. Arbour Stephens, relates an experience which raises an important question of principle. In the course of his duties as factory surgeon he recognised one of the workers, a young man, as the subject of syphilitic ulceration of the throat. A year later he learned that this man was about to be married. Thereupon, in addition to a communication made to the father, he telephoned to the "head priest" of the church of which the intended bride was a member and "told him the tale." From the fashion in which Dr. Stephens makes this announcement it is evident that no question of the propriety of his action has entered his mind. He was anxious, as he tells us, to prevent the propagation of syphilitic children, and with this laudable aim before him he took measures which lay ready to his hand. No one, we are sure, will doubt his good intentions, and his immediate purpose is likely to engage general sympathy. Further, he can quote in his support some recent and imposing expressions of opinion. In the view of a Royal Commission, a doctor, in circumstances such as those described by Dr. Stephens, ought to be free, and without risk of legal consequences, to warn a *fiancée* or her parents or guardians of the risks which her intended marriage involves; and this position has been endorsed by more than one organ of professional opinion. It has, obviously, a taking quality, and there are instances, let it freely be allowed, in which such a practice would have a protective value for the individuals concerned. Nevertheless, we venture to doubt whether all the consequences of acceptance of such a claim have been fully appreciated by those who support it. Particular cases may probably be stated in which a doctor's silence has meant cruel wrong and suffering. But, proverbially, hard cases make bad law, and the only sound basis for a general rule is to be derived from a consideration of its action over the whole area of its incidence.

The present position is that such a communication as is here in issue could only be made at the risk of an action for libel or slander. Doubtless, were it proved beyond question that the intending bridegroom was the subject of venereal disease in an infective stage, he, as plaintiff, would receive swift justice from a British jury. But, as would certainly sometimes occur, there might be differences of opinion on the point, and these differences would certainly find expression in the witness-box. In such circumstances the doctor, as defendant, might readily find himself in an awkward position, for a plaintiff who could show loss of reputation, and perhaps also loss of a bride and a fortune, might well press successfully for heavy damages. The doctor might have acted in all good faith and from the highest motives. But these pleas would not give him legal protection. The new proposal is that under the conditions here contemplated he shall enjoy such protection—that, in other words, a communication made by him for the purpose of protecting a woman from marriage with a man venereally infective shall be regarded as privileged. Given that he has acted honestly, with reasonable care, and without malice, the man concerned would not succeed against him in an action for libel or slander. Such a proposal clearly raises the whole question of professional secrecy, and it cannot be fully argued in a brief article. But there are two preliminary considerations which may suitably be weighed before the suggestion is summarily adopted, and these we will try to state in a few words.

The first is that the demand is a very delicate one, and that, once granted, it is almost certain to extend in area. At present it is limited by the qualification "venereally infective"; but is it possible to believe that if the principle is once accepted this qualification can be maintained? The limiting clause itself, though precise in form, will certainly receive conflicting interpretations. And, if professional secrecy is justly to be sacrificed for the purpose of protecting a woman from a primary infection, must it not equally be sacrificed to save a bride from a partner who may shortly become impotent, or blind, or insane? All of these are possibilities of a syphilitic infection. The woman and her friends are ignorant of these risks. But the man's doctor knows them. How can he be silent here when he has been taught that in the earlier stage of venereal infections it is his duty to intervene? Once leave well-defined ground and announce that a medical practitioner has, in his professional capacity, duties other than those which he owes to his patient, and it is certain the burden of such duties will rapidly be increased. Yet, again, let it be remembered that what is first defined as a legal right is very apt in time to be construed as a legal duty, and the doctor who abstains from doing what the law says he *may* do, will ere long find himself condemned for wilful neglect. In short, the present proposal will in practice involve something wider than its terms suggest.

Our second point is that the desired end can be quite surely attained without compelling the doctor to act as an informer against the man who has trusted him. Let a proposal for marriage be treated as a proposal for life insurance—that is, by an independent medical examination and a full report. Here is a method ready to hand, straightforward, and simple. It is more certain than the proposal of the Royal Commission; and with its universal adoption it would involve no moral slur. If the community will not take this obvious way of safety, why should the doctors be placed in a painful and invidious position?

